# Predicting School Grades: Can Conscientiousness Compensate for Intelligence?

**DOI:** 10.3390/jintelligence11070146

**Published:** 2023-07-20

**Authors:** Teresa Sophie Friedrich, Astrid Schütz

**Affiliations:** 1 Institute for Employment Research, 90478 Nuremberg, Germany; teresa.friedrich@iab.de; 2 Institute for Psychology, University of Bamberg, 96047 Bamberg, Germany

**Keywords:** intelligence, conscientiousness, school grades, LMS, gender

## Abstract

Intelligence and noncognitive factors such as conscientiousness are strongly related to academic performance. As theory and research differ with respect to their interplay in predicting performance, the present study examines whether conscientiousness compensates for intelligence or enhances the effect of intelligence on performance in 3775 13th grade students from Germany. Latent moderation analyses show positive main effects of intelligence and conscientiousness on grades. Further, analyses reveal synergistic interactions in predicting grades in biology, mathematics, and German, but no interaction in predicting grades in English. Intelligence and grades are more strongly linked if students are conscientious. Multigroup models detected gender differences in biology, but no differences with respect to SES. In biology, conscientiousness has especially strong effects in intelligent men. Conscientiousness thus enhances the effect of intelligence on performance in several subjects.

## 1. Introduction

Good grades in high school and university are an important starting point for a successful life. Grades determine whether students receive their preferred study or training place, and thus whether they can pursue their desired profession. Furthermore, grades predict university dropout (Behr et al. 2020) and are related to salary (Roth and Clarke 1998), job performance (Roth et al. 1996), and life satisfaction (Ng et al. 2015). However, how do students achieve good grades?

Intelligence is an important positive predictor of academic performance (e.g., Roth et al. 2015). However, in addition to intelligence, various socioemotional skills, such as subject-specific interests (Schiefele et al. 1993), self-concept (Huang 2011), self-efficacy (Multon et al. 1991), grit (Lam and Zhou 2021), or the Big Five personality traits, have an impact on academic performance.

Conscientiousness is especially relevant, as it is the strongest predictor of academic performance among the Big Five (Poropat 2009). According to Lechner et al. (2017), conscientiousness accounts for approximately as much variance in grades as intelligence does. It facilitates learning, as conscientious individuals are particularly ambitious, organised, reliable, hard-working, persistent, and disciplined (Bergold and Steinmayr 2018; Dumfart and Neubauer 2016). To summarize, both intelligence and conscientiousness influence academic performance, but do the two constructs interact in their influence on performance?

### 1.1. The Interplay of Intelligence and Conscientiousness in Predicting Performance

The effects of intelligence and conscientiousness on performance could take various forms. First, it is possible that intelligence and conscientiousness have independent effects on performance. Second, intelligence and conscientiousness might reinforce each other as a synergistic interaction, such that intelligent individuals particularly benefit from being conscientious. Third, the effect could be compensatory, such that less intelligent people benefit particularly from being conscientious.

The intelligence compensation hypothesis supports the idea of compensation, and suggests that less intelligent individuals become particularly conscientious, i.e., organised, thorough, persistent, and systematic, to compensate for a lack of intelligence. In contrast, intelligent individuals do not need to improve their conscientiousness because they can rely on their intelligence to master most tasks (Moutafi et al. 2003, 2004). Some studies found a negative correlation between intelligence and conscientiousness in line with the intelligence compensation hypothesis (e.g., Moutafi et al. 2003; Rammstedt et al. 2016), but most studies and meta-analytical results indicate no significant correlations (e.g., Ackerman and Heggestad 1997). For example, in a recent meta-analysis, Anglim et al. (2022) concluded that intelligence and conscientiousness are not correlated overall. However, they found negative correlations between intelligence and conscientiousness facets that focus on organization and order, and positive correlations with the competence facet.

Many studies that investigated the relationship between intelligence and conscientiousness did not test their relation to academic performance, and less attention has been given to possible interactions (Bergold and Steinmayr 2018). To our knowledge, seven studies thus far have investigated the interaction of intelligence and conscientiousness in predicting academic performance in twelve independent samples (Beaujean et al. 2011; Bergold and Steinmayr 2018; Brandt and Lechner 2022; Di Domenico and Fournier 2015; Meyer et al. 2022; Zhang and Ziegler 2015; Ziegler et al. 2009). These studies operationalized academic performance either with GPA/other grades or achievement tests. They included participants from fourth grade through university. The results showed a stronger interplay between intelligence and conscientiousness in predicting grades than in predicting achievement tests. All studies that operationalized performance via GPA/other grades showed evidence of synergy (Bergold and Steinmayr 2018; Di Domenico and Fournier 2015; Meyer et al. 2022; Ziegler et al. 2009): intelligent individuals in particular benefited from being conscientious. Most studies that operationalized performance via achievement tests showed no interaction (Beaujean et al. 2011; Brandt and Lechner 2022; Zhang and Ziegler 2015). However, Meyer et al. (2022) investigated four different performance indicators (grades, final exams, achievement tests, and GPA) and found synergistic interactions for all indicators. Furthermore, Ziegler et al. (2009) additionally found a compensatory interaction between intelligence and the conscientiousness facet achievement striving in predicting GPA in low performers.

The added value of the present paper is that we distinguish subjects and consider additional constructs: we calculate multigroup models for gender and SES. Our analyses thus provide new insights into whether the interplay of intelligence and conscientiousness in their effect on performance differs by gender or SES.

### 1.2. The Role of School Subjects

The demands and learning required vary by subject. Furthermore, the impact of personality traits and cognitive abilities on performance is context specific (e.g., Brandt et al. 2020): Brandt et al. (2020) observed a stronger association of both intelligence and conscientiousness with performance in mathematics than in German. Thus, to investigate the effect of intelligence and conscientiousness on performance, it is important to distinguish subjects. Of the previously mentioned studies with moderation analysis, only Meyer et al. (2022) considered grades in multiple (school) subjects. They found no significantly different interactions for German, mathematics, and English. In our analyses, we additionally consider biology. In contrast to mathematics, German, and English, biology is a subject that requires particularly good preparation. For biology tests, students have to memorize facts and schemes. By contrast, in math tests, students calculate problems. In German and English tests in upper secondary school, students mostly write essays or interpret texts. Thus, biology requires more diligence and preparation—as a consequence, conscientiousness should be important, which is why the subject is especially interesting when investigating possible compensation effects.

### 1.3. The Role of Gender

Conscientiousness (e.g., Donnellan and Lucas 2008; Schmitt et al. 2008), as well as school and university grades (e.g., Voyer and Voyer 2014), systematically differ between men and women. Boys dedicate less of their cognitive potential to school performance than girls (Spinath et al. 2010), and gender differences in grades can at least partially be explained by self-discipline and conscientiousness. Achievement tests underpredict the grades of girls and overpredict those of boys. Mediation analyses have shown that self-discipline explains at least partial grade differences between boys and girls (Duckworth and Seligman 2006). It is possible that the interplay of intelligence and conscientiousness also differs between boys and girls. Thus, we performed the analyses separately by gender. Some of the other studies included gender as a covariate in their models (e.g., Brandt and Lechner 2022; Meyer et al. 2022), and observed that women had better grades in languages/higher reading test scores, and men had better grades in mathematics/higher mathematic test scores. Brandt and Lechner (2022) further found higher reading competence gains for girls, and higher mathematics competence gains for boys. We extend that approach in our analyses by conducting multigroup models. Thus, we examine whether the interaction of intelligence and conscientiousness differs between women and men, rather than just controlling for gender.

### 1.4. The Role of SES

In addition to gender, SES is related to academic performance, but its interplay with intelligence and conscientiousness has not been analysed. We want to address this gap in the present study. High-SES students are more at risk of experiencing intergenerational downwards mobility than low-SES students, especially if their intelligence is low. Low-SES students already achieve upwards mobility when attending upper secondary school. In addition, if SES is very low, it cannot decline much more. Therefore, we expect students who are below average in intelligence, but high in SES, to try exceptionally hard and be especially conscientious. Von Stumm (2017) found that high-SES students perform better in school than low-SES students, even when they are less intelligent. Furthermore, conscientiousness and measures of SES correlated positively (Bucciol et al. 2015). However, one can also argue the other way around: low-SES students, especially if they are of at least medium intelligence, make a special effort because they are highly motivated to improve their standard of living. As with gender, some of the studies controlled for SES (e.g., Brandt and Lechner 2022; Meyer et al. 2022). Meyer et al. (2022) did not report specific results on SES, but Brandt and Lechner (2022) found that high-SES students had higher baseline test scores in reading and mathematics, and higher competence gains, than low-SES students. Therefore, we consider multigroup analyses examining different interactions of intelligence and conscientiousness in high- and low-SES students to be of particular interest.

### 1.5. Present Study

Intelligence is an important predictor of academic performance, but noncognitive factors such as conscientiousness also have an effect on performance. Can conscientiousness compensate for intelligence, or do the two constructs support each other? In other words, do intelligent people benefit more from being conscientious than less intelligent people, or is it the other way round?

Theoretical reasoning, specifically the intelligence compensation hypothesis (Moutafi et al. 2003, 2004), proposes that compensation is possible. However, empirical evidence is ambiguous and partly contrary to this argument. Meta-analyses that investigated the correlation between intelligence and conscientiousness showed negative correlations for some conscientiousness facets, but not a clear overall positive or negative correlation. Interaction analyses indicate that there may be synergistic effects when performance was operationalized with grades, but no interaction when performance was operationalized with achievement tests. In addition, previous research leaves some questions open: only one study has differentiated between grades across school subjects (Meyer et al. 2022), although the relationship of conscientiousness and intelligence with performance may vary with subject. Furthermore, no study has considered gender and SES via multigroup analyses, although there are gender differences in conscientiousness and grades, and SES is also related to grades.

The present study aims to close these research gaps by examining the interplay of intelligence and conscientiousness in predicting academic performance and considering gender, SES, and school subject. The aim is to shed light on the divergence between theory and research, and therefore to clarify whether conscientiousness can compensate for intelligence or enhance the effect of intelligence on performance.

**Hypothesis** **1.** *Intelligence has a positive effect on academic performance (grades) in all subjects (German, mathematics, English, and biology); we assume the strongest effect for mathematics grades.*

This is in line with Brandt et al. (2020) and B. Roth et al. (2015). Mathematics performance strongly relies on basic cognitive abilities (Rohde and Thompson 2007).

**Hypothesis** **2.** *Conscientiousness has a positive effect on grades in all subjects. We assume that the strongest effect will be found for biology and mathematics grades.*

Students need to study and memorize subject matter to receive excellent biology grades. For mathematics, continuous practice is necessary for performance (Meyer et al. 2019).

**Hypothesis** **3.** *Intelligence is negatively correlated with conscientiousness.*

This is in line with the intelligence compensation hypothesis: people may compensate for a lack of intelligence by being conscientious (Moutafi et al. 2003, 2004). The present empirical evidence does not suggest an overall negative correlation between intelligence and conscientiousness, but a recent meta-analysis showed negative correlations between intelligence and the conscientiousness facets of order and self-discipline. The items used in the present study focus on self-discipline; therefore, a negative correlation is to be expected.

**Hypothesis** **4.** *The intelligence compensation hypothesis (Moutafi et al. 2003, 2004) suggests a compensatory interaction between intelligence and conscientiousness in predicting grades. However, empirical evidence points to a synergistic interaction when performance is operationalized through grades. We therefore formulate competing hypotheses:*)

**Hypothesis** **4a.** *There is a compensatory interaction between intelligence and conscientiousness in predicting grades in all subjects beyond the main effects of either variable.*

**Hypothesis** **4b.** *There is a synergistic interaction between intelligence and conscientiousness in predicting grades in all subjects beyond the main effects of either variable.*^1^ *We expect the strongest interaction effects for biology and mathematics.*

Students need to memorize subject matters and practice continuously to perform well in these subjects. Thus, biology and mathematics grades offer the most opportunities for improvement based on conscientiousness.

**Hypothesis** **5.** *We assume stronger interactions for women than for men.*

In previous research, women achieved better grades than predicted based on their intelligence because of their higher conscientiousness scores (e.g., Kling et al. 2013).

**Hypothesis** **6a.** *We assume stronger interaction terms for above-average SES students than for below-average SES students.*

**Hypothesis** **6b.** *We assume stronger interaction terms for below-average SES students than for above-average SES students.*

On the one hand, high-SES students are at risk of downwards mobility; on the other hand, intelligent, but low-SES students may be highly motivated to improve their status.

## 2. Methods

### 2.1. Design and Sample

We analysed secondary data from the sixth wave of the study “Educational Outcomes of Students from Vocational and Academic Upper Secondary School” (LISA 6; Kampa et al. 2020b). This study examined 13th grade students in the 2012/2013 school year in the German federal state of Schleswig-Holstein. The sample covered all vocational upper secondary schools, as well as 17 out of the 99 academic upper secondary schools in Schleswig-Holstein (Leucht and Köller 2016).^2^

School coordinators completed student participation lists for all 13th grade students in their school with information on age, gender, and grades in selected subjects. Thus, complete data are available for these variables. Furthermore, the students participated in mandatory achievement tests and completed a voluntary student questionnaire. The study took place in the classroom and was supervised by trained test administrators (Leucht and Köller 2016). The codebook documents the exact wording and central item parameters (Kampa et al. 2020a). The data were made available by the Research Data Centre at the Institute for Educational Quality Improvement (FDZ at IQB).

The sample included 3775 students,^3^ of whom 54.82% were women. The mean age was 19.90 years, 15.10% had a migration background, and the average SES was 55.75. SES was coded using the Highest International Socio-Economic Index of Occupational Status (HISEI; Ganzeboom et al. 1992), with scores ranging between 12 and 89. The students who answered items on conscientiousness differed from those who did not respond to these items regarding gender (χ^2^(1) = 15.15, *p* < .001, w = 0.06), school type (χ^2^(1) = 4.12, *p* = .0423, w = 0.03), intelligence (*t*(3773) = −2.98, *p* = .0029, d = 0.10), and German grade (*t*(3769) = −2.66, *p* = .0079, d = 0.09). Women, students at vocational track schools, students with better German grades, and more intelligent students were more likely to provide these data. However, the effects were rather small or negligible in size.

### 2.2. Measures

*Intelligence*: The study examined intelligence via two subscales (V3 word analogies and N2 figure analogies) of the KFT 4-12+ R (Heller and Perleth 2000). A total of 19.7% of the academic track students and 12.7% of the vocational track students were absent on the day of the test. Missing values were handled via multiple imputation, and the dataset contains estimates for all 3775 students. The reliability for the intelligence measure (both subscales combined) in the sample of all 3775 students is 0.80 (reliability estimate retrieved from Leucht and Köller 2016).

*Conscientiousness*: Students answered four items from the BFI-K (Rammstedt and John 2005) to measure the Big Five personality trait conscientiousness: (1) “I complete tasks thoroughly”, (2) “I am comfortable; tend to be lazy”, (3) “I am efficient and work fast”, and (4) “I make plans and carry them out”. Answers ranged from 1 “*very inappropriate*” to 5 “*very appropriate*”. The student questionnaire was voluntary; only 1714 students responded to the conscientiousness items; Cronbach’s alpha in the present sample is 0.67 (*N* = 1620).

*Academic performance*: We used German, mathematics, English, and biology report card grades from 13th grade students to measure academic performance. In Germany, upper school grades range between 0 “*insufficient*” and 15 “*excellent*” points. Table 1 and Table 2 present descriptives of and correlations between the main variables.

*Control variables*: We controlled for age, gender, migration background (first and second generation), type of school (vocational vs. academic upper secondary school), students’ intentions to go to university, academic self-concept, and motivation for and interest in German, mathematics, English, and science.

*Student’s intention to attend university*: Following Trautwein et al. (2007), students were asked if they wanted to attend university after school. Answers ranged from 1 “*certainly not*” to 4 “*for sure*”.

*Academic self-concept*: Academic self-concept was assessed with four items from Schwanzer et al. (2005) on a four-point scale from 1 “*not at all true*” to 4 “*totally true*”: (1) “I am sure in advance that I will not be able to solve many exercises, because I am not talented with this matter”, (2) “I wish I was as intelligent as the others”, (3) “I often think I’m not as smart as the others”, (4) “Compared to others, I am not that talented”. The reliability in the present sample is 0.83 (*N* = 2033).

*Motivation/Interest*: Four revised items from Baumert et al. (1997), Kunter et al. (2002), and Trautwein et al. (2007) measured motivation for or interest in German, mathematics, English, and science: (1) “I like to know a lot in the subject of xy”, (2) “I would like to have more xy classes than I have now”, (3) “I am looking forward to a lesson in xy”, and (4) “xy is important to me personally”. Scales ranged from 1 “*not at all true”* to 4 *“totally true”*. The reliability is 0.88 for German (*N* = 2025), 0.91 for mathematics (*N* = 2045), 0.81 for English (*N* = 2041), and 0.90 for science (*N* = 2000).

### 2.3. Statistical Analyses

We analysed the research question by moderated regression analyses. As the presence of measurement error is especially problematic for measuring multiplicative and nonlinear effects such as interactions, SEM is particularly valuable for investigating these analyses (Little et al. 2006). The latent moderated structural equations (LMS) approach (Klein and Moosbrugger 2000) provides an unbiased and efficient method for implementing latent interactions in structural equation models.

We first estimated four regression models with German, mathematics, English, and biology grades as dependent variables, and intelligence and conscientiousness as independent variables. Next, we included the interaction terms as additional parameters in the models. Thus, we can investigate whether the interaction term accounts for a significant amount of variance beyond intelligence and conscientiousness. As the chi^2^ difference test and the resulting fit statistics are not suitable for nonlinear latent variable models (Klein and Schermelleh-Engel 2010), we compared the two models via the log-likelihood ratio test, as described in Maslowsky, Jager, and Hemken (Maslowsky et al. 2015).

Next, we estimated multigroup models with men and women and below-average SES and above-average SES students.^4^ To compare the relations of latent variables over groups, there must be weak measurement invariance (Christ and Schlüter 2012). For a more intuitive interpretation of the data, and because of collinearity, we standardized intelligence and conscientiousness before the analyses (Aiken et al. 1991).

We considered the multilevel structure of the data (TYPE = COMPLEX), as well as missing values (FIML), in all analyses. Furthermore, we controlled for gender, age, migration background, type of school, study intention, academic self-concept, and motivation in all final models, as these constructs (particularly the latter two) are strongly related to intelligence and conscientiousness. Analyses were performed in R (R Core Team 2020) and Mplus (Muthén and Muthén 1998).

## 3. Results

Both intelligence and conscientiousness had positive effects on grades in German, mathematics, English, and biology classes. To investigate differences in estimates between subjects, we examined whether the 95% confidence intervals overlapped. Intelligence had significantly stronger effects on grades in mathematics than on those in other subjects. Conscientiousness had further stronger effects on grades in mathematics than in English and German, but not in biology. There was no significant correlation between intelligence and conscientiousness. The models explained 8.22% of the variance in German grades, 18.44% of the variance in mathematics grades, 7.89% of the variance in English grades, and 12.03% of variance in biology grades. All four models fit the data well (CFI ≥ 0.963, TLI ≥ 0.930, RMSEA ≤ 0.036, SRMR ≤ 0.026).

**Figure 1:**
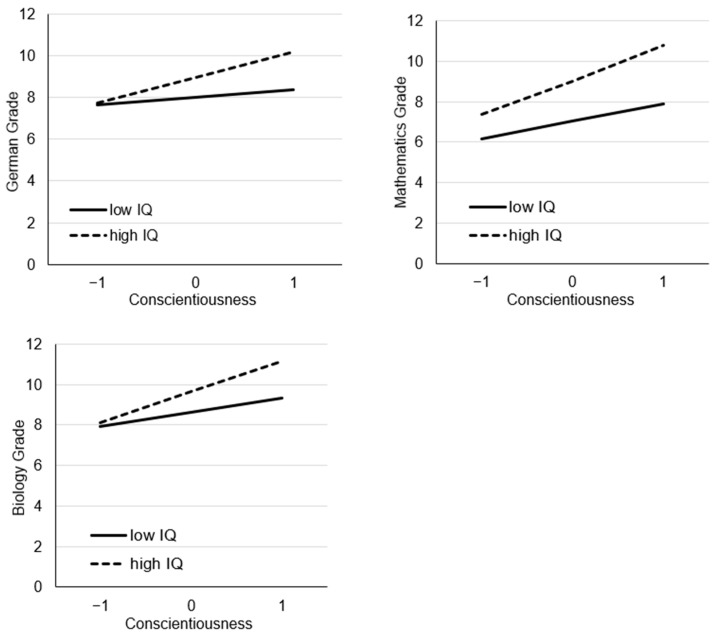
Simple slopes for high- and low-intelligence students.

All models with the interaction term fit the data significantly better than the models without the interaction term (German χ^2^_diff, df=1_ = 22.40, *p* < .001, mathematics χ^2^_diff, df=1_ = 14.55, *p* < .001, English χ^2^_diff, df=1_ = 5.14, *p* = .0234, biology χ^2^_diff, df=1_ = 10.64, *p* = .0011). R^2^ was 0.1037 for German grade, 0.1926 for mathematics, 0.0833 for English, and 0.1281 for biology grades. The interaction terms explained additional 2.15% of variance in German grades, additional 0.82% of variance in mathematics, additional 0.44% of variance in English, and additional 0.78% of variance in biology grades. The interaction terms provide important information beyond that provided by models that do not include interaction. For German, mathematics, and biology grades, we found a synergistic interaction; for English grades, we did not find a significant interaction. The interaction terms hardly differed between the subjects, and the 95% confidence intervals overlapped; nevertheless, the interaction was largest in mathematics, followed by German and biology. Figure 1 shows the simple slopes for the interaction analyses. To show the robustness of the results, we ran additional manifest regression analyses with the 1714 students who answered the conscientiousness items. The results did not differ between manifest and latent analyses; only the effect sizes were slightly smaller in the manifest analyses (see Table A3).

**Figure 2:**
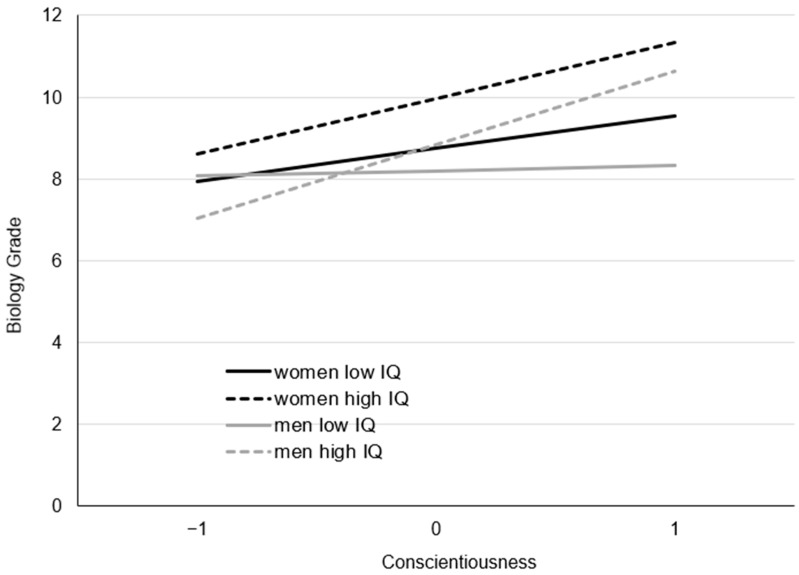
Simple slopes for high and low intelligent men and women in biology.

Next, we analysed the multigroup models for gender and SES to investigate their effect on the interplay of intelligence and conscientiousness when predicting academic performance. We found partial weak measurement invariance across groups (gender χ^2^_diff, df=2_ = 1.14, *p* = .565, SES χ^2^_diff, df=2_ = 3.91, *p* = .142).^5^ In German, mathematics, and English, the interaction terms did not differ between men and women. In biology, men and women differed significantly in their interaction term: while we still found a synergistic interaction for men, the interaction term for women was no longer significant. Figure 2 shows simple slopes in biology for men and women.

Regarding SES, below-average and above-average SES students did not differ in their interaction of intelligence and conscientiousness while predicting grades. The models with control variables provide a comparable picture with smaller differences. Table A1 provides more detailed information.

## 4. Discussion

As in previous studies, and in line with our hypotheses, we found larger positive effects of intelligence and conscientiousness on grades in science than on grades in languages (e.g., Brandt et al. 2020). One possible explanation is that students have to memorize subject matter in science classes, and therefore invest effort and learning time. Especially for mathematics, it is necessary to repeat and practice calculations for good grades. Furthermore, the word and figure analogies subscales of the KFT capture logical reasoning, which is also particularly helpful in science classes.

Contrary to our hypothesis, intelligence did not correlate significantly with conscientiousness. The state of research is ambiguous at this point. However, a recent meta-analysis (Anglim et al. 2022) showed no overall correlation between intelligence and conscientiousness, but only with specific conscientiousness facets. It is possible that the correlation between intelligence and conscientiousness is highly dependent on the sample, operationalization of conscientiousness, and situation.

Furthermore, we found significant synergistic interactions in German, mathematics, and biology. This is in contrast to the intelligence compensation hypothesis, but in line with the results of previous research on grades (e.g., Bergold and Steinmayr 2018; Di Domenico and Fournier 2015; Meyer et al. 2022; Ziegler et al. 2009). Intelligent students benefit particularly when they are conscientious, but a compensatory effect in less intelligent students is less pertinent. It is possible that students need to be conscientious to use their full intelligence potential and that, in turn, at least some intelligence is needed to benefit from conscientiousness.

Meyer et al. (2022) converges with the present study. They examined the interaction of intelligence and conscientiousness in predicting various performance indicators in the school subjects German, mathematics, and English, and found the same results. In adding to these findings and other previous studies, we calculated multigroup models with gender and SES: we found no differences between groups with respect to SES, and only differences in biology interaction terms between men and women. For men with lower scores on the intelligence test, it made no difference whether they were conscientious or not. Intelligent men, on the other hand, performed significantly better if they were rather conscientious. In contrast, women, regardless of their intelligence level, performed better when they were rather conscientious. The results were contrary to our hypothesis, as we had expected stronger interaction effects in women. It is possible that conscientious women at all intelligence levels strive to optimise their performance, while in men it is the intelligent ones in particular who strive for performance. Furthermore, women are typically more interested in biology, which may be why women, unlike men, work hard at all levels of intelligence and do the best they can.

Why did we not find any compensatory interaction? On the one hand, intelligence did not correlate significantly with conscientiousness in our sample. It would be interesting for future studies to investigate whether a compensatory interaction can be found in samples with a negative correlation between intelligence and conscientiousness. On the other hand, the intelligence compensation hypothesis may be wrong. To date, only correlative studies support the intelligence compensation hypothesis; regression analyses with interaction terms between intelligence and conscientiousness tend to speak against compensation. However, these studies—as well as the present study—only cross-sectionally examined the extent to which the relationship between conscientiousness and performance varies with the level of intelligence. According to the intelligence compensation hypothesis, however, individuals become more conscientious to compensate for a lack of intelligence; change in conscientiousness is at the focus of the theory. The existing studies examined only the possible effects of an underlying process in which conscientiousness compensates for lack of intelligence, but did not look at the process itself, or at increases in conscientiousness. Thus, further analyses with longitudinal data are needed.

### Limitations

After the present analysis was completed, a paper was published that converges with the present paper and relies on the same dataset (Meyer et al. 2022). This increases our confidence in the results, but reduces their novelty. Nevertheless, in the present paper, we additionally examine the school subject of biology, and analyse multigroup models with gender and SES—which goes beyond that paper.

In addition, our sample included only students who attended an upper secondary school in Schleswig-Holstein. Upper secondary schools are the highest school track in Germany, and successful completion leads to a university entrance qualification. On the one hand, it is questionable whether the results can be transferred to other German federal states or other countries. On the other hand, upper secondary schools place high demands on their students, so that only particularly high-achieving students can attend. It can be assumed that the mean and variance in intelligence and conscientiousness in our sample therefore differ from those in the general population.

Participation in the questionnaire was voluntary, which is why many students did not participate. The students who did not answer the items on conscientiousness differed significantly from others regarding gender, intelligence, type of school, and grade in German.

Furthermore, intelligence measurements in studies should generally be treated cautiously, as the results do not have any consequences for the participants. It is likely that conscientious participants will try harder and take the test more seriously, which would lead to better results (Chamorro-Premuzic and Furnham 2004). It is possible that this effect offsets the negative correlation between intelligence and conscientiousness hypothesised by the intelligence compensation hypothesis. This may be the reason we did not find a significant relationship between intelligence and conscientiousness in the present study.

## 5. Conclusions

Our results support previous empirical findings on a synergy between intelligence and conscientiousness, rather than the intelligence compensation hypothesis: we found no correlation between intelligence and conscientiousness, but synergistic interactions between the two constructs in predicting German, mathematics, and biology grades. Gender differences were found for biology: Intelligent men particularly benefitted from being conscientious, whereas women benefit from conscientiousness regardless of their intelligence level. Thus, especially for men, conscientiousness is important to exploit the person’s full cognitive potential. In any case, we found positive effects of both intelligence and conscientiousness on grades. Both traits help students to achieve good grades.

## Figures and Tables

**Table 1:** Descriptives of the Main Variables.

	M	SD	Min	Max	Skew	N
Intelligence	0	0.67	−3.02	2.22	−0.27	3775
Conscientiousness	3.65	0.69	1	5	−0.37	1714
German grade	8.47	2.35	2	15	0.24	3771
Mathematics grade	8.05	3.08	1	15	0.09	3771
English grade	8.65	2.64	1	15	0.1	3765
Biology grade	9.13	9.13	2	15	−0.04	2369

**Table 2:** Correlations Between all Variables.

	1	2	3	4	5	6	7	8	9	10	11	12	13	14	15	16	17	18	19
1. Intelligence																			
2. Conscientiousness	−0.04																		
3. German grade	**0.20**	**0.20**																	
4. Mathematics grade	**0.33**	**0.26**	**0.42**																
5. English grade	**0.23**	**0.14**	**0.61**	**0.40**															
6. Biology grade	**0.21**	**0.27**	**0.53**	**0.54**	**0.47**														
7. Age	**−0.14**	**−0.05**	**−0.12**	**−0.19**	**−0.14**	**−0.13**													
8. Female	**−0.15**	**0.27**	**0.11**	0.01	**0.06**	**0.07**	**−0.11**												
9. Migration background	**−0.16**	−0.02	**−0.13**	**−0.10**	**−0.06**	**−0.08**	**0.16**	−0.03											
10. Academic track school	**−0.37**	**0.09**	**−0.15**	**−0.13**	**−0.16**	−0.02	**0.27**	**0.06**	**0.10**										
11. Study intention	**0.13**	**0.12**	**0.26**	**0.21**	**0.23**	**0.23**	−0.02	**−0.06**	**0.07**	**−0.13**									
12. Self-concept	**0.21**	**0.21**	**0.23**	**0.25**	**0.24**	**0.22**	−0.04	**−0.21**	**−0.09**	−0.02	**0.18**								
13. Motivation German	**−0.12**	**0.18**	**0.36**	**−0.14**	**0.10**	**0.05**	0.01	**0.21**	0.00	0.04	0.05	−0.03							
14. Motivation mathematics	**0.24**	**0.26**	**0.07**	**0.54**	**−0.06**	**0.25**	−0.02	**−0.15**	0.00	0.02	**0.16**	**0.15**	**−0.10**						
15. Motivation English	0.03	**0.16**	**0.25**	0.01	**0.57**	**0.12**	**−0.05**	**0.05**	0.03	**−0.06**	**0.15**	**0.06**	**0.30**	**−0.17**					
16. Motivation science	**0.14**	**0.19**	**0.12**	**0.15**	−0.00	**0.38**	**0.06**	**−0.09**	0.01	−0.03	**0.17**	**0.08**	0.04	**0.39**	−0.03				
17. Openness	0.01	**0.11**	**0.15**	**−0.09**	**0.11**	0.04	**0.07**	**0.21**	**0.09**	−0.02	**0.10**	−0.01	**0.31**	**−0.10**	**0.21**	0.05			
18. Extraversion	**−0.10**	**0.17**	**0.17**	−0.01	**0.13**	0.07	−0.03	**0.04**	0.01	−0.01	**0.09**	**0.24**	**0.15**	**−0.09**	**0.13**	**−0.08**	**0.11**		
19. Agreeableness	**−0.07**	0.06	0.04	**0.06**	−0.01	0.04	**−0.11**	**0.20**	−0.05	0.01	−0.02	0.02	**0.07**	**0.07**	0.01	0.04	0.06	0.08	
20. Neuroticisms	**−0.07**	−0.01	0.06	−0.04	0.04	0.00	0.00	**0.29**	0.06	0.04	−0.04	**−0.43**	**0.13**	**−0.08**	0.05	**0.05**	**0.19**	**−0.36**	**−0.14**

*Note*. Numbers in bold are significant at *p* < .05.

## Data Availability

The study used secondary data which were made available by the Research Data Centre at the Institute for Educational Quality Improvement (FDZ at IQB). http://doi.org/10.5159/IQB_LISA_6_v1.

## Notes

1 We added hypothesis 4b after preregistration. The hypotheses at preregistration were based solely on theory. As the state of research suggested different outcomes than theory, we added an additional hypothesis.

2 The academic upper secondary schools in the sample already participated in previous waves of the LISA survey. The sample was drawn as a multistage stratified cluster sample prior to the first survey. In wave six, the sample comprises 18.6% of all students at academic upper secondary schools in Schleswig-Holstein (Leucht and Köller 2016).

3 We ran robustness checks with the 1714 students who completed the conscientiousness items. We found no major differences as compared with the results in the full sample. See Table A2 for the results.

4 As the individuals with below-average and above-average SES did not differ in their interaction terms, we did not analyse differences between combined gender and SES groups, in deviation from the preregistered analyses.

5 For both gender and SES, the factor loading for one item was freed (“I am comfortable; tend to be lazy”).

## Appendix A

**Table A1:** Moderated Regression Analyses Predicting School Grades.

	**M1**		**M2**		**M3**	
	**β**	**95% CI**	**β**	**95% CI**	**β**	**95% CI**
	**German**					
Intercept	8.48 ***	[8.30, 8.65]	8.48 ***	[8.30, 8.66]	8.59 ***	[8.21, 8.96]
Intelligence	0.47 ***	[0.38, 0.56]	0.47 ***	[0.38, 0.56]	0.37 ***	[0.26, 0.48]
Conscientiousness	0.73 ***	[0.53, 0.93]	0.79 ***	[0.61, 0.97]	0.25 **	[0.12, 0.38]
IQ × C			0.42 ***	[0.27, 0.57]	0.34 ***	[0.19, 0.50]
Female					0.48 ***	[0.28, 0.67]
Age					−0.05	[−0.19, 0.10]
Migration background					−0.52 ***	[−0.71, −0.32]
Academic track school					−0.33	[−0.79, 0.13]
Study intention					0.46 ***	[0.37, 0.55]
Self-concept					0.73 ***	[0.52, 0.94]
Motivation					1.01 ***	[0.90, 1.13]
	**M1**		**M2**		**M3**	
	**β**	**95% CI**	**β**	**95% CI**	**β**	**95% CI**
	**Mathematics**					
Intercept	8.05 ***	[7.89, 8.22]	8.05 ***	[7.89, 8.22]	8.00 ***	[7.64, 8.37]
Intelligence	1.04 ***	[0.91, 1.16]	1.02 ***	[0.90, 1.14]	0.53 ***	[0.39, 0.67]
Conscientiousness	1.27 ***	[1.05, 1.49]	1.28 ***	[1.07, 1.49]	0.44 ***	[0.28, 0.60]
IQ × C			0.42 ***	[0.22, 0.63]	0.23 *	[0.06, 0.41]
Female					0.59 ***	[0.34, 0.85]
Age					−0.38 ***	[−0.55, −0.21]
Migration background					−0.36	[−0.66, −0.05]
Academic track school					−0.19	[−0.60, 0.22]
Study intention					0.32 ***	[0.19, 0.45]
Self-concept					0.74 ***	[0.53, 0.94]
Motivation					1.64 ***	[1.50, 1.77]
	**M1**		**M2**		**M3**	
	**β**	**95% CI**	**β**	**95% CI**	**β**	**95% CI**
	**English**					
Intercept	8.65 ***	[8.47, 8.83]	8.65 ***	[8.47, 8.84]	8.54 ***	[8.18, 8.89]
Intelligence	0.61 ***	[0.52, 0.70]	0.61 ***	[0.52, 0.70]	0.45 ***	[0.34, 0.56]
Conscientiousness	0.65 ***	[0.43, 0.87]	0.66 ***	[0.45, 0.86]	0.03	[−0.17, 0.24]
IQ × C			0.25	[0.03, 0.46]	0.22 *	[0.05, 0.40]
Female					0.51 ***	[0.31, 0.70]
Age					−0.17	[−0.32, −0.02]
Migration background					−0.12	[−0.40, 0.16]
Academic track school					−0.17	[−0.59, 0.25]
Study intention					0.29 ***	[0.20, 0.39]
Self-concept					0.82 ***	[0.63, 1.02]
Motivation					1.59 ***	[1.46, 1.73]
	**M1**		**M2**		**M3**	
	**β**	**95% CI**	**β**	**95% CI**	**β**	**95% CI**
	**Biology**					
Intercept	9.12 ***	[8.90, 9.34]	9.13 ***	[8.90, 9.35]	8.33 ***	[7.93, 8.73]
Intelligence	0.51 ***	[0.36, 0.67]	0.50 ***	[0.35, 0.65]	0.46 ***	[0.28, 0.63]
Conscientiousness	1.13 ***	[0.87, 1.38]	1.10 ***	[0.85, 1.34]	0.53 ***	[0.30, 0.75]
IQ × C			0.41 *	[0.13, 0.69]	0.33 *	[0.09, 0.57]
Female					0.64 **	[0.31, 0.98]
Age					−0.26 *	[−0.45, −0.07]
Migration background					−0.13	[−0.49, 0.22]
Academic track school					0.62 *	[0.12, 1.12]
Study intention					0.42 ***	[0.30, 0.55]
Self-concept					0.58 ***	[0.34, 0.81]
Motivation					0.95 ***	[0.81, 1.09]

*Note*. * *p* < .05, ** *p* < .01, *** *p* < .001.

**Table A2:** **Robustness Check**—Moderated Regression Analyses Predicting School Grades With 1714 Students Who Completed Conscientiousness Items.

	**M1**		**M2**		**M3**	
	**β**	**95% CI**	**β**	**95% CI**	**β**	**95% CI**
	**German**					
Intercept	8.59 ***	[8.31, 8.82]	8.59 ***	[8.36, 8.82]	8.59 ***	[8.23, 8.95]
Intelligence	0.44 ***	[0.31, 0.57]	0.47 ***	[0.38, 0.56]	0.35 ***	[0.23, 0.47]
Conscientiousness	0.72 ***	[0.53, 0.91]	0.74 ***	[0.57, 0.91]	0.25 **	[0.12, 0.38]
IQ × C			0.32 **	[0.14, 0.50]	0.35 ***	[0.19, 0.51]
Female					0.49 ***	[0.27, 0.71]
Age					−0.04	[−0.18, 0.10]
Migration background					−0.51 ***	[−0.73, −0.28]
Academic track school					−0.32	[−0.78, 0.13]
Study intention					0.45 ***	[0.35, 0.55]
Self-concept					0.74 ***	[0.52, 0.96]
Motivation					1.01 ***	[0.87, 1.15]
	**M1**		**M2**		**M3**	
	**β**	**95% CI**	**β**	**95% CI**	**β**	**95% CI**
	**Mathematics**					
Intercept	8.15 ***	[7.93, 8.37]	8.15 ***	[7.93, 8.37]	7.96 ***	[7.57, 8.35]
Intelligence	1.11 ***	[0.94, 1.28]	1.11 ***	[0.94, 1.28]	0.53 ***	[0.38, 0.68]
Conscientiousness	1.28 ***	[1.06, 1.50]	1.30 ***	[1.09, 1.51]	0.45 ***	[0.29, 0.61]
IQ × C			0.39 **	[0.18, 0.60]	0.23 *	[0.05, 0.41]
Female					0.57 ***	[0.30, 0.84]
Age					−0.40 ***	[−0.58, −0.22]
Migration background					−0.37*	[−0.66, −0.07]
Academic track school					−0.16	[−0.58, 0.26]
Study intention					0.31 ***	[0.17, 0.45]
Self-concept					0.79 ***	[0.57, 1.01]
Motivation					1.61 ***	[1.48, 1.75]
	**M1**		**M2**		**M3**	
	**β**	**95% CI**	**β**	**95% CI**	**β**	**95% CI**
	**English**					
Intercept	8.73 ***	[8.51, 8.94]	8.73 ***	[8.51, 8.95]	8.52 ***	[8.17, 8.87]
Intelligence	0.68 ***	[0.57, 0.78]	0.68 ***	[0.57, 0.78]	0.44 ***	[0.32, 0.57]
Conscientiousness	0.62 ***	[0.41, 0.83]	0.64 ***	[0.43, 0.84]	0.03	[−0.18, 0.23]
IQ × C			0.21 *	[0.04, 0.38]	0.22 *	[0.05, 0.40]
Female					0.55 ***	[0.35, 0.75]
Age					−0.17	[−0.32, −0.02]
Migration background					−0.11	[−0.39, 0.17]
Academic track school					−0.18	[−0.59, 0.23]
Study intention					0.29 ***	[0.18, 0.39]
Self-concept					0.86 ***	[0.65, 1.07]
Motivation					1.58 ***	[1.43, 1.73]
	**M1**		**M2**		**M3**	
	**β**	**95% CI**	**β**	**95% CI**	**β**	**95% CI**
	**Biology**					
Intercept	9.16 ***	[8.89, 9.43]	9.17 ***	[8.90, 9.44]	8.31 ***	[7.90, 8.72]
Intelligence	0.56 ***	[0.38, 0.74]	0.55 ***	[0.38, 0.73]	0.47 ***	[0.29, 0.65]
Conscientiousness	1.16 ***	[0.90, 1.42]	1.15 ***	[0.89, 1.41]	0.54 ***	[0.32, 0.77]
IQ × C			0.39 *	[0.10, 0.68]	0.34 *	[0.08, 0.60]
Female					0.66 **	[0.31, 1.01]
Age					−0.26	[−0.49, −0.04]
Migration background					−0.19	[−0.55, 0.18]
Academic track school					0.64 *	[0.13, 1.16]
Study intention					0.42 ***	[0.29, 0.55]
Self-concept					0.56 ***	[0.32, 0.80]
Motivation					0.87 ***	[0.70, 1.03]

*Note*. * *p* < .05, ** *p* < .01, *** *p* < .001.

**Table A3:** Robustness Check—Regression Analyses with Observed Variables.

	**M1**		**M2**	
	**β**	**95% CI**	**β**	**95% CI**
	**German**			
Intercept	8.59 ***	[8.50, 8.68]	8.59 ***	[8.50, 8.68]
Conscientiousness	0.46 ***	[0.36, 0.55]	0.45 ***	[0.36, 0.54]
Intelligence	0.45 ***	[0.35, 0.55]	0.45 ***	[0.36, 0.55]
IQ × C			0.17 **	[0.06, 0.27]
	**M1**		**M2**	
	**β**	**95% CI**	**β**	**95% CI**
	**Mathematics**			
Intercept	8.15 ***	[8.04, 8.36]	8.16 ***	[8.04, 8.27]
Conscientiousness	0.74 ***	[0.63, 0.86]	0.74 ***	[0.63, 0.86]
Intelligence	1.12 ***	[1.00, 1.24]	1.13 ***	[1.01, 1.25]
IQ × C			0.19 *	[0.07, 0.32]
	**M1**		**M2**	
	**β**	**95% CI**	**β**	**95% CI**
	**English**			
Intercept	8.73 ***	[8.63, 8.82]	8.73 ***	[8.63, 8.83]
Conscientiousness	0.37 ***	[0.29, 0.49]	0.39 ***	[0.29, 0.49]
Intelligence	0.68 ***	[0.58, 0.78]	0.68 ***	[0.58, 0.79]
IQ × C			0.10	[−0.02, 0.21]
	**M1**		**M2**	
	**β**	**95% CI**	**β**	**95% CI**
	**Biology**			
Intercept	9.18 ***	[9.06, 9.31]	9.19 ***	[9.07, 9.32]
Conscientiousness	0.67 ***	[0.54, 0.79]	0.66 ***	[0.53, 0.78]
Intelligence	0.58 ***	[0.44, 0.71]	0.57 ***	[0.43, 0.71]
IQ × C			0.25 *	[0.09, 0.41]

*Note*. Sample sizes are: N_D_ = 1713, N_M_ = 1713, N_E_ = 1707, N_B_ = 1039; R^2^ for M1 and M2 are: R^2^_D0_ = 0.073; R^2^_D1_ = 0.078; R^2^_M0_ = 0.179; R^2^_M1_ = 0.183; R^2^_E0_ = 0.088; R^2^_E1_ = 0.089; R^2^_B0_ = 0.107; R^2^_B1_ = 0.116. * *p* < .05, ** *p* < .01, *** *p* < .001.
